# Formulation and Characterization of Captopril-Loaded Chitosan Mucoadhesive Buccal Films with Different Permeation-Enhancing Components

**DOI:** 10.3390/pharmaceutics18081015

**Published:** 2026-08-16

**Authors:** Hala Rayya, Raghad Alsheikh, Dániel Nemes, Lajos Nagy, Géza Regdon, Ildikó Bácskay, Krisztián Pamlényi, Katalin Kristó

**Affiliations:** 1 Institute of Pharmaceutical Technology and Regulatory Affairs, University of Szeged, Eötvös u. 6., 6720 Szeged, Hungary; rayya.hala@stud.u-szeged.hu (H.R.); regdon.geza.laszlo@szte.hu (G.R.J.); kristo.katalin@szte.hu (K.K.); 2 Department of Pharmaceutical Technology, Faculty of Pharmacy, University of Debrecen, 4002 Debrecen, Hungary; alsheikh.raghad@pharm.unideb.hu (R.A.); nemes.daniel@pharm.unideb.hu (D.N.); bacskay.ildiko@pharm.unideb.hu (I.B.); 3 Doctorate School of Pharmaceutical Sciences, University of Debrecen, 4002 Debrecen, Hungary; 4 Department of Applied Chemistry, Institute of Chemistry, Faculty of Science and Technology, University of Debrecen, 4032 Debrecen, Hungary; nagy.lajos@science.unideb.hu; 5 Institute of Healthcare Industry, University of Debrecen, 4032 Debrecen, Hungary

**Keywords:** buccal delivery, captopril, chitosan ascorbate, mucoadhesive film, permeation enhancer, TR146 cells

## Abstract

**Background/Objectives:** The buccal mucosa offers a promising non-invasive route for systemic drug delivery, particularly for hydrophilic compounds like captopril (CAP), which exhibit low permeability and are subject to gastrointestinal instability and first-pass metabolism. This study aimed to develop and characterize captopril-loaded, chitosan-based mucoadhesive buccal films with different permeation enhancers and to evaluate their physicochemical properties, drug release, cytocompatibility, and in vitro transport across a TR146 buccal epithelial cell model. **Methods:** Films were prepared by the solvent-casting method using chitosan as the film-forming polymer. Different enhancers were investigated, including organic acid salts of chitosan (ascorbate, citrate, and lactate) and chemical permeation enhancers (sodium lauryl sulfate, polyethylene glycol 400, Span 20, and EDTA). **Results:** The resulting films exhibited acceptable thickness, moisture content, appropriate mechanical properties, and good mucoadhesive strength. In vitro dissolution studies demonstrated rapid CAP release, with >50% released within 15 min and near-complete release by 180 min across all formulations. Cytotoxicity assessment via a Neutral Red uptake assay in TR146 cells confirmed high cell viability (>81%) after 4 h of exposure, indicating good biocompatibility. In vitro permeation experiments revealed that films prepared with chitosan ascorbate and chitosan lactate enhanced CAP transport compared to other formulations, achieving the highest flux and apparent permeability coefficients. **Conclusions:** These findings demonstrate that chitosan ascorbate and lactate salts effectively improve the buccal permeability of captopril while maintaining good film properties and biocompatibility. This work highlights the potential of chitosan ascorbate- and lactate-based mucoadhesive films as an efficient platform for the buccal delivery of CAP.

## 1. Introduction

The buccal route of drug administration has emerged as an attractive, non-invasive alternative to conventional oral delivery, particularly for compounds that suffer from extensive first-pass metabolism, gastrointestinal instability, or variable absorption in the gastrointestinal tract [1]. The buccal mucosa is richly vascularized and drains directly into the jugular vein, enabling rapid systemic absorption while avoiding hepatic metabolism and gastrointestinal degradation [2]. Additionally, the buccal mucosa is easily accessible, allowing easy self-administration and the flexibility to remove the dosage form immediately if adverse effects occur or treatment needs to be interrupted [3]. It also offers better patient compliance than many other transmucosal (rectal, vaginal, intranasal, etc.) or parenteral routes, as it avoids the discomfort associated with injections and the variability caused by food intake or gastrointestinal motility [4].

Despite these advantages, the buccal epithelium, a non-keratinized stratified squamous tissue, functions as a permeability barrier. Intercellular lipids and cell membranes restrict the transport of hydrophilic or ionized molecules, often resulting in suboptimal absorption for many therapeutic agents [5]. To overcome this limitation, formulation strategies such as the incorporation of chemical permeation enhancers and the use of mucoadhesive polymers have been extensively investigated [6]. These approaches aim to transiently modulate the barrier properties without causing irreversible damage or irritation to the mucosal tissue.

Mucoadhesive buccal films represent a promising dosage form for drug delivery across the buccal mucosa. These films provide direct contact with the absorption site, resist salivary clearance, and allow unidirectional drug release toward the mucosa while minimizing swallowing and systemic side effects [7]. Among the film-forming polymers used, chitosan (CHI)**,** a cationic polysaccharide derived from chitin, is particularly advantageous. It exhibits good biocompatibility, biodegradability, and inherent mucoadhesive properties through ionic interactions with negatively charged sialic acid residues in mucin glycoproteins [8,9]. Furthermore, CHI possesses intrinsic permeability-enhancing effects by transiently opening tight junctions via interactions with cell membrane components, facilitating paracellular transport of hydrophilic drugs [10].

Captopril (CAP), a hydrophilic angiotensin-converting enzyme inhibitor (ACEI), is used for the management of hypertension, heart failure, and left ventricular dysfunction after myocardial infarction [11]. It is absorbed after oral administration, with a bioavailability of approximately 60–75% (reduced by food intake), reaching peak plasma concentrations within about 1 h. Its elimination half-life is short—approximately 2 h—necessitating multiple daily dosing, primarily due to renal elimination [12]. CAP is also a relevant model drug for studying buccal permeability enhancement due to its physicochemical properties. It exhibits high aqueous solubility and low lipophilicity, characterized by a log P of approximately 0.34 [13,14]. Although some classifications list captopril as BCS Class I, it is frequently regarded in buccal and oral film research as a BCS Class III compound [15,16], in which high solubility is accompanied by low permeability, making permeability the rate-limiting step for absorption. This profile, in addition to its low molecular weight of 217.29 g/mol, renders CAP particularly suitable for assessing formulation strategies aimed at improving buccal mucosa transport.

Chemical permeation enhancers are commonly incorporated to further increase transbuccal transport of hydrophilic drugs, such as CAP [17]. These agents, including surfactants, bile salts, fatty acids, chelators, and others, act via mechanisms such as lipid extraction or disruption, protein interaction, or tight junction modulation, thereby increasing paracellular or transcellular flux [18,19]. Their effects must be balanced against potential mucosal irritation or cytotoxicity, particularly in repeated applications. Thus, the challenge lies in achieving significant enhancement with minimal cytotoxicity or mucosal irritation, as excessive barrier disruption can compromise safety.

Reliable preclinical evaluation of such systems requires a validated in vitro model that closely mimics human buccal conditions. The TR146 cell line, derived from a human buccal carcinoma, forms a stratified and non-keratinized epithelium when cultured on permeable supports and is a well-validated in vitro model for mechanistic studies of buccal permeability and enhancer safety [20]. It correlates with ex vivo human and porcine buccal mucosa data for permeability assessment [20,21].

The aim of the current study was to develop and comprehensively characterize chitosan mucoadhesive buccal films of various compositions loaded with captopril. The prepared films were evaluated for their physicochemical and mechanical properties, mucoadhesive performance, drug loading, in vitro drug release, chemical compatibility, cytocompatibility, and transport of captopril through the TR146 buccal epithelial cell model. In vitro transport testing was also performed to compare the performance of the developed formulations in a cellular buccal model.

The formulations contained different chitosan salt forms, including chitosan lactate (CHI-Lac), chitosan citrate (CHI-Cit), and chitosan ascorbate (CHI-Asc), as well as conventional formulation excipients, such as EDTA, Span 20, polyethylene glycol (PEG) 400, and sodium lauryl sulfate (SLS). Captopril was used as a hydrophilic model drug to evaluate the performance of the developed buccal film formulations. Particular attention was paid to the differences in captopril transport observed among the tested formulations, while the underlying mechanisms of these differences were investigated beyond the scope of this study. The results provide a comprehensive evaluation of chitosan-based buccal films and identify formulation compositions associated with differences in captopril transport under the experimental conditions used.

## 2. Materials and Methods

### 2.1. Materials

Medium molecular weight CHI (average MW: 1250 kDa; batch number: 889EMW) (≥90% degree of deacetylation) was used as a film-forming polymer (Glentham Life Sciences Ltd., Corsham, UK). Glycerol (GLY) (Ph.Eur.9.0; ≥99.5%), added to the film as a plasticizer, was supplied by Merck (Darmstadt, Germany). Ascorbic acid (Ph.Eur.9.0; Lot No. D19408696), citric acid (Ph.Eur.8.0; batch number: BX1994654), and lactic acid, added to the film to dissolve CHI and enhance permeation, were purchased from Molar Chemicals Ltd. (Halásztelek, Hungary). SLS, Span 20 (sorbitan monolaurate, TCI Chemicals, Tokyo, Japan), ethylenediaminetetraacetic acid disodium salt dihydrate (EDTA) (EDTA·Na_2_·2H_2_O, batch number: 04082301, Spektrum 3D, Debrecen, Hungary), and PEG 400 (Ph.Eur.9.0; Lot No. EC1418626, Molar Chemicals Ltd., Halásztelek, Hungary) were incorporated as chemical permeation enhancers. CAP (Ph.Eur.9.0; Lot No. 460500723) was obtained as a gift from EGIS Pharmaceuticals PLC (Budapest, Hungary). Acetic acid (Ac) (min. 99.8%, Sigma-Aldrich, Darmstadt, Germany) provided an appropriate acidic medium for CHI dissolution during the preparation of the films with the non-acidic permeation enhancers. Mucin type II from the porcine stomach dispersion (10 *w*/*w* %), used in the in vitro mucoadhesion test, was purchased from Sigma-Aldrich (St. Louis, MO, USA). Distilled water was used as a solvent in the preparation of the solutions.

### 2.2. Preparation of Buccal Films

Polymeric films were prepared using the solvent-casting method. The compositions of the formulated polymer film solutions are presented in Table 1. The required amount of CHI was dispersed in an aqueous acidic medium and stirred using an overhead stirrer (DLS, VELP Scientifica, Usmate, Italy) equipped with a propeller-type mixing rod at 100 rpm until uniform dispersion was achieved. The dispersion was then allowed to stand for approximately four hours at room temperature to ensure complete dissolution of the polymer and for the air bubbles to disappear from the homogeneous solution. Once a clear solution was obtained, the calculated amounts of CAP and the penetration enhancer were added and mixed thoroughly. Subsequently, the plasticizer, GLY, was incorporated into the solution and stirred for an additional 20 min. A predetermined volume of the final solution was poured into plastic Petri dishes (9 cm in diameter) and dried in a ventilated oven (Memmert GmbH + Co. KG, Schwabach, Germany) under controlled conditions (temperature: 30 °C; airflow: 30% fan capacity). After drying, the films were carefully sealed until further evaluation.

### 2.3. Film Thicknesses

Film thickness was measured using a micrometer screw gauge (measurement range: 0–25 mm; resolution: 0.001 mm; Mitutoyo Co., Ltd., Kanagawa, Japan). Measurements were taken at five different positions on each film, and the mean thickness values along with the corresponding standard deviations were calculated.

### 2.4. Moisture Content

The moisture content of the films was determined using a halogen moisture analyzer (MAC 50/NH, RADWAG, Radom, Poland). For each formulation, three parallel measurements were carried out at a drying temperature of 105 °C using the automatic switch-off mode. The obtained values were expressed as percentages.

### 2.5. Breaking Hardness

The breaking hardness of the films was determined using a texture analyzer developed at our institute [22]. The instrument is equipped with interchangeable sample holders and probes designed for various mechanical tests. For hardness measurement, the film sample was fixed in the holder at the base of the device, and a needle-shaped probe (3 mm in diameter) was driven downward toward the film at a constant speed of 20 mm/min. The probe movement was terminated upon film rupture, and both the breaking force (0–50 N) and the test duration were recorded. The data were displayed as force–time curves using the software connected to the analyzer. Each formulation was tested in triplicate, and the results were expressed as mean values ± standard deviation.

### 2.6. Folding Endurance

Folding endurance was evaluated manually by repeatedly folding a film sample with a defined surface area of 4 cm^2^ along the same axis. The folding endurance value was determined by recording the number of folds the film could withstand before tearing occurred. Each formulation was tested in triplicate.

### 2.7. Puncture Resistance Time

Puncture resistance time was determined from the force–time curves obtained during the breaking hardness test. The time interval from the initial contact of the needle with the film to complete puncture was used as an indirect measure of puncture resistance time and expressed in seconds. Each formulation was evaluated in triplicate, and the results were reported as mean values ± standard deviation.

### 2.8. In Vitro Mucoadhesion Test

The in vitro mucoadhesion test was performed using the same texture analyzer equipped with appropriate accessories and test parameters. A rod-shaped probe (9 mm in diameter) served as the sample holder, and the film was attached to the lower surface of the probe using double-sided adhesive tape. A freshly prepared mucin dispersion (10% *w*/*w*; 25 µL) was uniformly spread onto a disc (11 mm in diameter) positioned at the base of the instrument. The probe was then lowered toward the mucin-coated disc, and a contact force of 30 ± 0.1 N was applied for 30 s to ensure intimate contact between the film and the mucin layer [23,24]. Subsequently, the probe was withdrawn at a constant speed, and the maximum detachment force was recorded as the peak of the force–time curve, representing the mucoadhesive force. Each formulation was tested in triplicate, and the results were expressed as mean values ± standard deviation.

### 2.9. Drug Content Uniformity

Drug-loaded films were prepared by incorporating CAP in amounts corresponding to a final drug content of 20 mg per 4 cm^2^ of film. To determine the captopril content, film samples (2 cm × 2 cm) were dissolved in 100 mL of phosphate buffer (pH 6.8) and stirred for 1 h to ensure complete drug extraction. The captopril concentration was then quantified using a UV–VIS spectrophotometer (Genesys 10S, Thermo Fisher Scientific, Waltham, MA, USA) at a wavelength of 208 nm. Quantification was performed using an external calibration curve over the concentration range of 0–30 µg/mL. The calibration equation was *y =* 0.0383*x* + 0.0088 (*R*^2^ = 0.9989), where *x* is the captopril concentration (µg/mL), and *y* is the absorbance. The calculated limit of detection (LOD) and limit of quantification (LOQ) were 1.1 and 3.4 µg/mL, respectively. All measurements were performed in triplicate (*n* = 3).

### 2.10. Dissolution Test

The dissolution test was performed using a six-station USP XXIV type II dissolution apparatus (paddle over disk) (Erweka DT700 LH, Langen, Germany) in accordance with pharmacopeia guidelines [25]. Phosphate buffer (500 mL, pH 6.8), maintained at 37 °C, was used as the dissolution medium, and the paddle rotation speed was set to 50 rpm. To ensure unidirectional drug release, a film sample (4 cm^2^) was fixed onto a glass slide using double-sided adhesive tape, after which the slide was immersed in the dissolution vessel. At predetermined time intervals (5, 10, 15, 20, 30, 45, 60, 90, 120, 150, and 180 min), 5 mL aliquots were withdrawn and analyzed using a UV–VIS spectrophotometer (Genesys 10S, Thermo Fisher Scientific, Waltham, MA, USA) at a wavelength of 208 nm. Each experiment was conducted in triplicate, and the results were expressed as mean values ± standard deviation. The release data were fitted to the zero-order, first-order, Higuchi, and Korsmeyer–Peppas kinetic models using linear regression analysis [26]. The model with the highest coefficient of determination (R^2^) was considered to provide the best description of the release behavior. For the Korsmeyer–Peppas model, only the initial stage of release (≤60% cumulative drug release) was used for analysis.

### 2.11. FT-IR Spectroscopy

Fourier transform infrared spectroscopy (FT-IR) was used to examine the possible interactions between the drug and the excipients in the prepared films. Analyses were performed on an Avatar 330 FT-IR spectrometer (Thermo Fisher Scientific Inc., Waltham, MA, USA) equipped with a Zn/Se horizontal attenuated total reflectance (HATR) accessory. The film samples were placed directly onto the crystals and scanned in the range of 600 to 4000 cm^−1^. Spectra were collected with a resolution of 32 cm^−1^ using 128 scans after applying H_2_O and CO_2_ corrections. The resulting spectra were analyzed using the SpectraGryph software (version 1.2.15, Dr. Friedrich Menges Software, Obersdorf, Germany).

### 2.12. Permeation and Cytotoxicity Test

#### 2.12.1. Cell Culturing

TR-146 cells (European Collection of Authenticated Cell Cultures, ECACC No. 10032305) were cultured in Dulbecco’s Modified Eagle Medium (DMEM) supplemented with 10% (*v*/*v*) fetal bovine serum (FBS), 1% (*v*/*v*) non-essential amino acids, 3.7 g/L sodium hydrogen carbonate, 100 IU/mL penicillin, and 100 μg/mL streptomycin sulfate. The cells were maintained at 37 °C in a humidified atmosphere containing 5% CO_2_ using standard plastic cell culture flasks. The culture medium was replaced regularly, and cells were subcultured upon reaching appropriate confluency. Cells between passages 20 and 28 were used for all experiments.

#### 2.12.2. Cytotoxicity Test

The cytotoxicity was evaluated using the Neutral Red (NR) uptake assay. TR146 cells were seeded into 96-well plates at a density of 10,000 cells per well and cultured for 7 days. Subsequently, film samples with an area of 7.06 mm^2^ were placed onto the cells and incubated for 2 or 4 h. Following exposure, the films were removed, and the cells were gently washed with phosphate-buffered saline (PBS). Thereafter, 100 µL of NR solution (33.3 mg/mL; Alfa Aesar, Karlsruhe, Germany) prepared in cell culture medium was added to each well, and the plates were incubated for an additional 2 h. The NR solution was then discarded, and 100 µL of an isopropanol–1 M hydrochloric acid mixture (25:1, *v*/*v*) was added to extract the incorporated dye. Blank wells were used for background correction. Absorbance was measured at 540 nm using a Thermo Fisher Multiskan GO microplate reader (Thermo Fisher Scientific, USA). Cell viability was determined relative to untreated control cells maintained in cell culture medium.

#### 2.12.3. In Vitro Permeation Test

Cells were seeded into ThinCert^®^ PET cell culture inserts (0.4 µm pore diameter, 2 × 10^6^/cm^2^ pore density on a 33.6 mm^2^ culturing surface) (Greiner BioOne, Mosonmagyaróvár, Hungary) and allowed to grow into a monolayer with the addition of fresh culture medium every 2 days for 2 weeks. The transepithelial electrical resistance (TEER) of the monolayer was measured regularly using a Millicell ERS 1 device (Merck, Budapest, Hungary). Only inserts with a resistance value over 130 Ω·cm^2^ were used for the permeation tests. The films were cut to an equal weight with a size of 7.06 mm^2^, and their CAP content was 0.3530 mg. Before the experiment, the culture medium was removed and replaced with 400 µL of Hank’s Balanced Salt Solution (initial pH: 7.4) (HBSS) in the apical compartment (AC) and 1000 µL of HBSS in the basolateral compartment (BC). The study was initiated by placing the film onto the apical side of the insert. At 20, 40, 60 and 80 min, 200 µL samples were collected from the BC and immediately replenished with an equal volume of fresh HBSS to maintain a constant volume throughout the experiment.

#### 2.12.4. Analytical Measurement and Calculation Based on Permeation Results

The samples were introduced into an LC/MS chromatographic system consisting of a Waters 2695 Separations Module with a thermostable autosampler (5 °C) and a column module (35 °C)—a VDSphere PUR 100 C18-M-SE reverse phase C-18 column (4.6 mm × 150 mm, 5 μm) (VDS Optilab Chromatographie Technik GmbH, Berlin, Germany) coupled with a MicroTOF-Q type Qq-TOF MS instrument (Bruker Daltonik, Bremen, Germany)—using an electrospray ion source (ESI) with the positive ion mode. The flow rate and the injected volume were 1.0 mL/min and 10 µL, respectively, in all cases. A splitter was applied between the HPLC and MS, obtaining a flow rate of 0.1 mL/min for the ESI ion source. The mass spectra were calibrated externally using the exact masses of the clusters generated from the electrosprayed solution of sodium trifluoroacetate (NaTFA). Eluent A was 0.1% trifluoroacetic acid (TFA) in water; eluent B was acetonitrile. The eluent table was as follows: 0 min and 5 min—80% A, 20% B; 15 min and 20 min—30% A, 70% B; 20 min and 25 min—80% A, 20% B. The recorded mass spectra were evaluated with the Bruker Data Analyzer 3.4 software.

Average flux values for the transport were calculated as follows:

(1) $$\frac{Q_{n}}{A\times \Delta t_{a}}=J$$

where *Q_n_* is the cumulative mass of transported API calculated for each sampling time in mg (considering the previously taken sample amounts for 40, 60 and 80 min), *A* is the surface area of the cell culture insert in cm^2^, and Δ*t* is the time between sampling in minutes.

Apparent permeability flux values for the transport were calculated as follows:

(2) $$\frac{\Delta Q}{A\times \Delta t\times C_{0}}=P_{app}$$

where Δ*Q* is the cumulative amount of transported API calculated for each sampling time in mol (considering the previously taken sample amounts for 40, 60 and 80 min), *A* is the surface area of the cell culture insert in cm^2^, Δ*t* is the time between sampling in seconds, and *C*_0_ is the initial concentration of the API in the apical compartment in mol/cm^3^.

## 3. Results and Discussion

**Figure 1:**
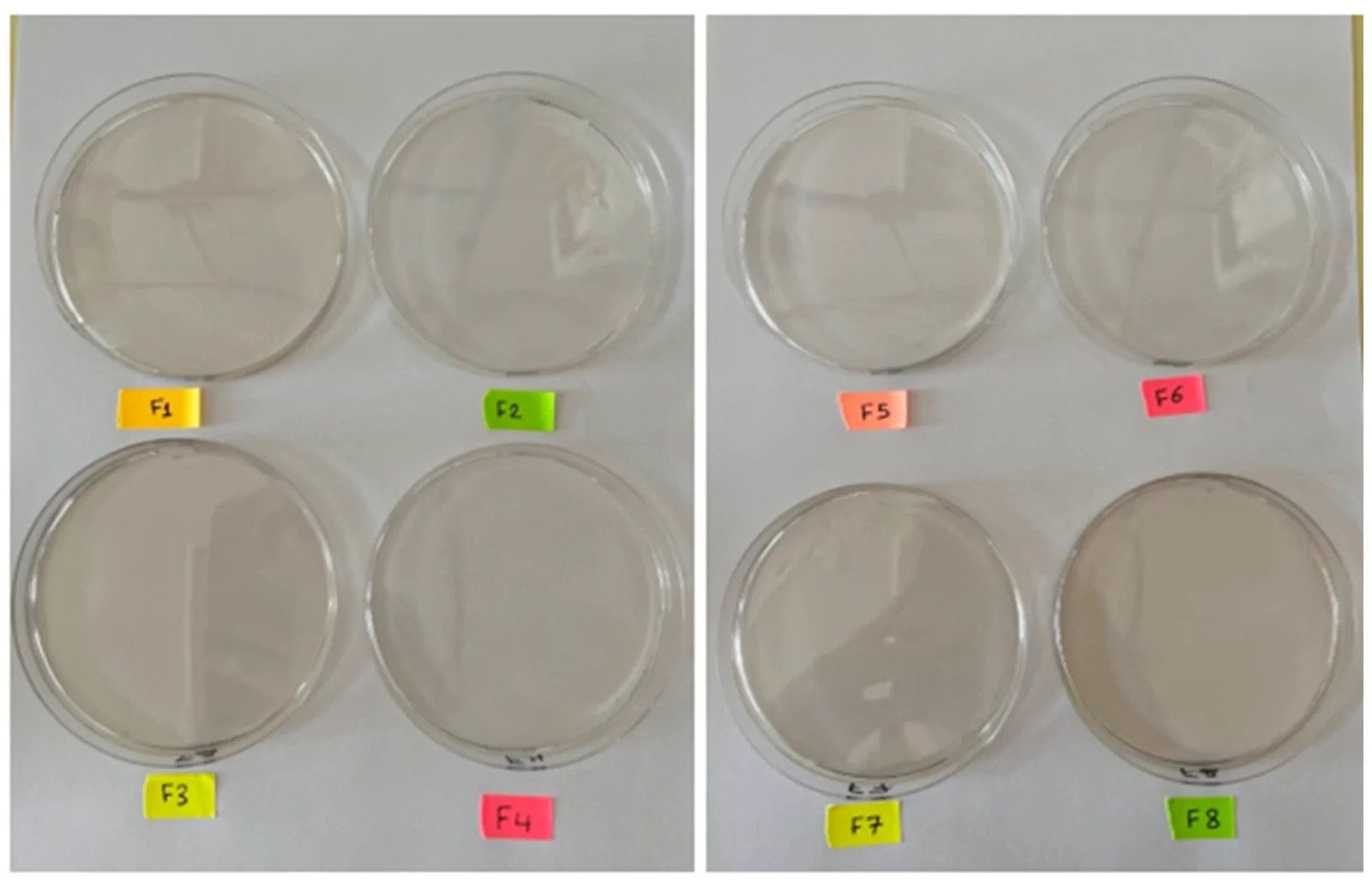
Photographs of the prepared captopril-loaded chitosan buccal film.

All prepared CHI-based buccal films were visually smooth, homogeneous, flexible, and free from visible air bubbles or cracks, indicating successful film formation using CHI as the primary polymer and GLY as the plasticizer. The films were transparent with no noticeable visual differences among the formulations (Figure 1). The incorporation of different permeation enhancers did not adversely affect the integrity of the films, confirming the suitability of the solvent-casting method for all formulations. The physicochemical properties of the prepared films, including thickness, moisture content, breaking hardness, mucoadhesion force, puncture resistance time, folding endurance, and drug content, are presented in Table 2.

### 3.1. Film Thickness

The thickness of the prepared CHI-based mucoadhesive films was measured using a micrometer, with values ranging from 164 µm (F4, CHI-SLS) to 270 µm (F2, CHI-Cit) across the formulations (Table 2). All films exhibited thicknesses within the typical range reported for buccal or mucoadhesive polymeric films (approximately 50–1000 µm), which ensures patient comfort upon application [27]. No clear correlation was observed between the type of acid or additive used and film thickness. For instance, films with different organic acids (ascorbic in F1, citric in F2, and lactic in F3) showed comparable thicknesses (228–270 µm), while acetic acid-based formulations (F4–F8) tended to be slightly thinner (164–187 µm). The incorporation of surfactants or enhancers, such as SLS (F4), Span 20 (F6), or EDTA (F7), did not produce significant differences in thickness relative to the control acetic acid film (F8, 173 µm). This suggests that the film-forming process and chitosan concentration (2 g/100 mL) were the primary determinants of thickness, with minor influences from the additives under the casting conditions employed. Thinner films, such as F4, may offer advantages in terms of flexibility and patient comfort, whereas slightly thicker films (e.g., F2) could provide enhanced mechanical protection or prolonged residence time. However, all formulations maintained thicknesses compatible with mucoadhesive performance, as evidenced by the accompanying mucoadhesion data. Overall, the thickness results confirm that the developed films possess suitable physical dimensions for buccal drug delivery applications.

### 3.2. Moisture Content

The moisture content of the films ranged from 3.63% to 8.41% (Table 2). The lowest moisture content value can be found in the case of the F1 film, which contains ascorbic acid. Films containing hydrophilic additives, such as PEG 400 (F5) and SLS (F4), exhibited higher moisture content. The relatively high moisture content observed in the case of F5 can be attributed to the pronounced hygroscopic nature of PEG 400, whose hydrophilic ether groups readily form hydrogen bonds with water molecules, thereby enhancing water uptake and retention within the chitosan-based polymeric matrix [28]. On the other hand, the elevated moisture content observed in F4 may be attributed to the ionic nature of SLS, which can enhance water affinity within the chitosan matrix and promote increased water retention through modifications in polymer–water interactions [29]. Controlled moisture content is crucial, as excessive water uptake can compromise mechanical strength, while insufficient moisture may reduce flexibility, patient comfort, and handling. However, all formulations remained within the acceptable moisture range (2–10%) reported for chitosan-based buccal films [23].

### 3.3. Breaking Hardness, Puncture Resistance Time, and Folding Endurance

The mechanical performance of buccal films is a critical quality attribute: the dosage form must withstand handling during packaging and application while remaining sufficiently flexible to adapt to the buccal mucosa without cracking or damaging. Thus, in the present study, breaking hardness, puncture resistance time, and folding endurance were evaluated. Breaking hardness ranged from 15.14 N (F2) to 48.17 N (F7) depending on the composition (Table 2), indicating that all films possessed sufficient mechanical strength for practical handling. In buccal systems, a breaking hardness value above 10 N is generally considered adequate to prevent mechanical damage during application [23]; all formulations exceeded this threshold. In addition, all formulations demonstrated adequate flexibility, as evidenced by high folding endurance values (>300 folds) (Table 2) [30]. This indicates that the films can withstand repeated handling without breaking, an essential requirement for buccal film systems. Puncture resistance time values indicated good elasticity for all formulations (Table 2), with PEG-400-containing films (F5) showing the highest puncture resistance time, confirming its role as an efficient plasticizer and permeability enhancer. PEG 400 is well known for increasing chain mobility and flexibility in polymeric drug delivery systems [31].

### 3.4. Mucoadhesive Properties

The results of the in vitro mucoadhesivity study are shown in Table 2. The highest mucoadhesive forces were recorded for F5 (18.42 N), F2 (16.67 N) and F1 (16.21 N), followed by F7 (13.76 N). Lower values were observed with F3 (11.23 N) and F4 (10.6 N). The superior mucoadhesion observed in F5 suggests a synergistic effect, whereby PEG 400, as a hydrophilic co-plasticizer, may improve chain mobility and interpenetration of chitosan into the mucin layer [31]. In contrast, the lower mucoadhesion force observed with SLS (F4) may result from surfactant-induced alterations in the mucus gel structure or competitive interactions at the interface, potentially reducing the availability of chitosan’s cationic sites for mucin binding [32]. Overall, the mucoadhesive performance of all formulations exceeded 7 N, especially the F1, F2 and F5 samples, which are considered sufficient for prolonged residence time on the buccal mucosa [33], supporting effective drug absorption.

### 3.5. Drug Content Uniformity

The results of the drug content uniformity measurement are shown in Table 2. The average drug content across formulations ranged from 17.4 mg (F5) to 22.2 mg (F7), corresponding to 87–111% of the theoretical target loading. Most formulations exhibited good uniformity, with low standard deviations (RSD < 5%). These results meet the suitable extent of content according to the European Pharmacopoeia (Ph.Eur.12) [34].

### 3.6. In Vitro Drug Release Studies

**Figure 2:**
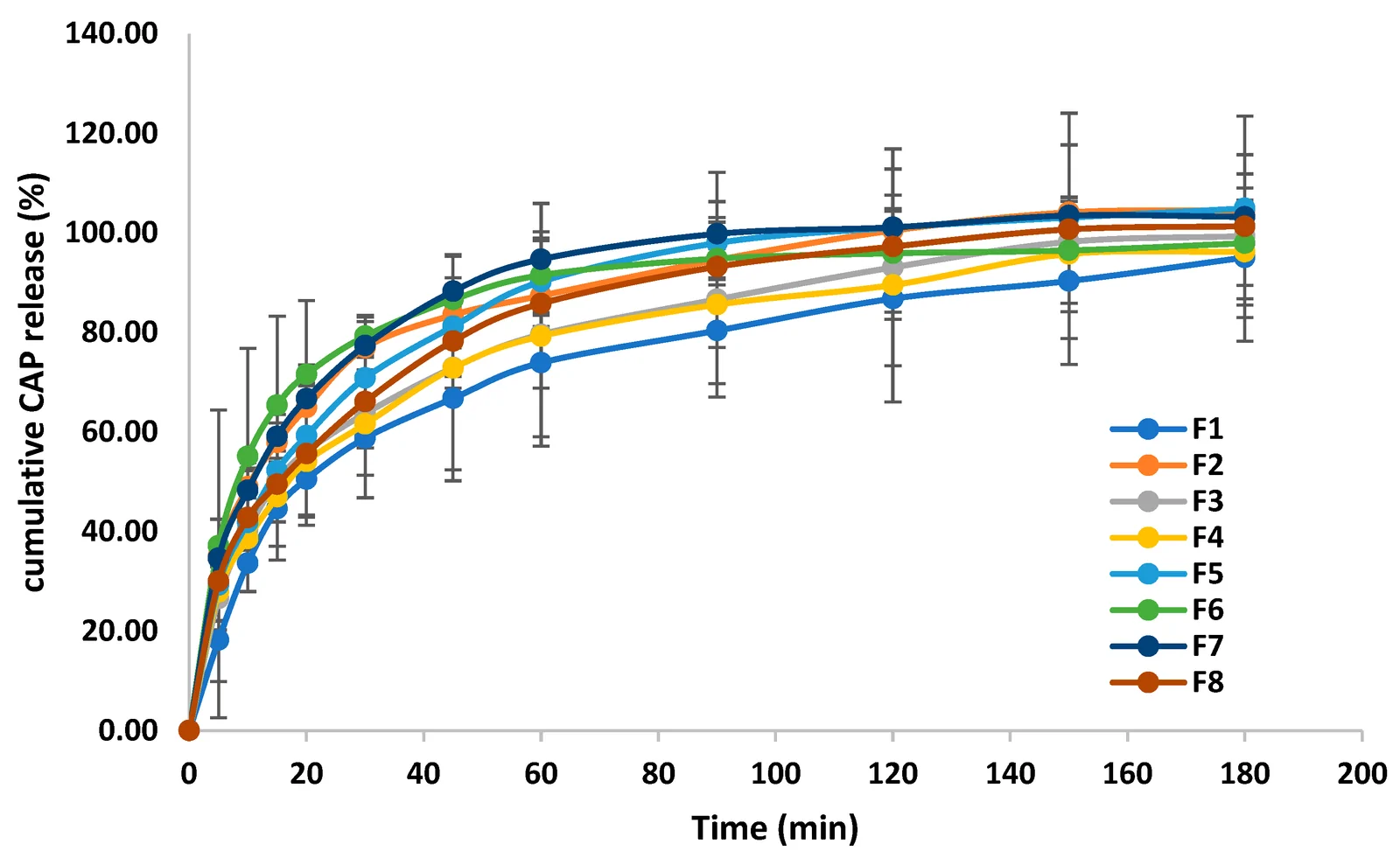
Cumulative CAP release (%) profiles from chitosan-based buccal films (mean ± SD, *n* = 3).

Figure 2 shows the drug release curves of different compositions. All formulations exhibited a rapid initial burst release, with more than 50% of the loaded CAP released within 15 min, followed by near-complete release by 180 min.

The cumulative drug release profiles revealed that all formulations achieved high drug release within 180 min under the tested conditions. The CAP release occurred in two stages: in the first stage, it was characterized by an initial burst phase (typically 5–15 min); in the second stage, it was followed by a more gradual phase. By 180 min, mean release exceeded 90% for all formulations. This release behavior aligns with the hydrophilic nature of the chitosan matrix, plasticized with glycerol, and the high aqueous solubility of CAP. This is further consistent with findings reported for captopril-loaded orally dispersible films based on polyvinyl alcohol, sodium alginate, and gelatin blends [35], which also demonstrated fast drug release, where the hydrophilic polymer matrix in both systems facilitates prompt hydration and diffusion.

However, notable differences emerged among the acid variants and additives. The Span-20-containing formulation (F6) exhibited the fastest initial release, with 66% of captopril released after 15 min, followed by the EDTA-containing film (F7, 59%) and the citric acid-containing film (F2, 58%). In contrast, the ascorbic acid formulation (F1) showed the slowest initial release, with only 45% drug release at 15 min. After 60 min, the EDTA-containing formulation (F7) demonstrated the highest cumulative release (95%), followed by the Span-20-containing film (F6, 92%) and the citric acid-containing film (F2, 87%). The ascorbic acid formulation (F1) continued to exhibit the lowest release (74%), while the remaining formulations showed intermediate release profiles.

The superior performance of Span 20 (F6) can be attributed to the non-ionic surfactant properties, which could reduce surface tension, improve the wetting of the polymeric matrix, and enhance water penetration into the film [36,37]. These actions facilitate rapid hydration, increase matrix porosity, and promote faster diffusion of captopril from the chitosan matrix. However, the SLS-containing film (F4) demonstrated moderate release performance, falling in the lower mid-range among the tested formulations. Despite SLS being a well-known anionic surfactant and permeation enhancer, its incorporation did not markedly accelerate captopril release. The citric acid-containing film (F2) demonstrated markedly faster CAP release than the chitosan ascorbate film (F1) and slightly higher release than the chitosan lactate film (F3). Since the formulations differed only in the acid used for chitosan solubilization, these findings indicate that the counterion plays an important role in determining the release behavior of the resulting chitosan salts.

The fastest release was observed from the chitosan citrate films (F2). The reason for this phenomenon is the differences in matrix hydration and drug diffusion arising from the distinct physicochemical properties of citrate compared with those of lactate and ascorbate. Conversely, the slower release from the chitosan ascorbate films (F1) suggests stronger retention of captopril within the polymer matrix, potentially resulting from differences in hydrogen-bonding interactions or polymer organization.

The PEG-400-containing film (F5) also showed accelerated early release, attributable to the hydrophilic plasticizer’s ability to increase matrix porosity, water uptake, and free volume within the polymer network. A similar enhancement in release rate due to PEG 400 has been reported in polymeric film systems [38] and also documented in polysaccharide-based film systems [39].

Overall, these results demonstrate that additive selection significantly influences the release kinetics of the drug in chitosan buccal films after 15 min. In the first 15 min, all compositions exhibited a burst-release phase.

To further investigate the release mechanism, the experimental data were fitted to the zero-order, first-order, Higuchi, and Korsmeyer–Peppas kinetic models (Table 3). Overall, the zero-order model provided the poorest fit for all formulations (R^2^ = 0.5901–0.7505), indicating that the CAP was not released at a constant rate from the chitosan films. The Higuchi model showed a moderate correlation (R^2^ = 0.766–0.931), suggesting that diffusion contributed to drug release but was not the sole controlling mechanism [40]. In contrast, the Korsmeyer–Peppas model exhibited the highest correlation coefficients for six of the eight formulations (R^2^ = 0.993–1.000), whereas the first-order model best described the release profiles of the CHI-Asc (F1) and CHI-Ac (F8) films. These findings indicate that the CAP release from most formulations was governed predominantly by diffusion through the hydrated polymeric matrix, with contributions from polymer relaxation, rather than by constant-rate release. The release exponent (n) obtained from the Korsmeyer–Peppas model ranged from 0.4448 to 0.6623. Formulations F2, F4, F7, and F8 exhibited n values below 0.50, indicating predominantly quasi-Fickian behavior. In the quasi-Fickian model, CAP was released from the films by two different mechanisms: swelling of the film and CAP diffusion through pores partially filled with liquid [41,42]. In contrast, F1, F3, F5, and F6 showed n values between 0.50 and 0.662, suggesting non-Fickian transport, in which both diffusion and polymer relaxation contribute to drug release [26].

### 3.7. FT-IR Analysis

**Figure 3:**
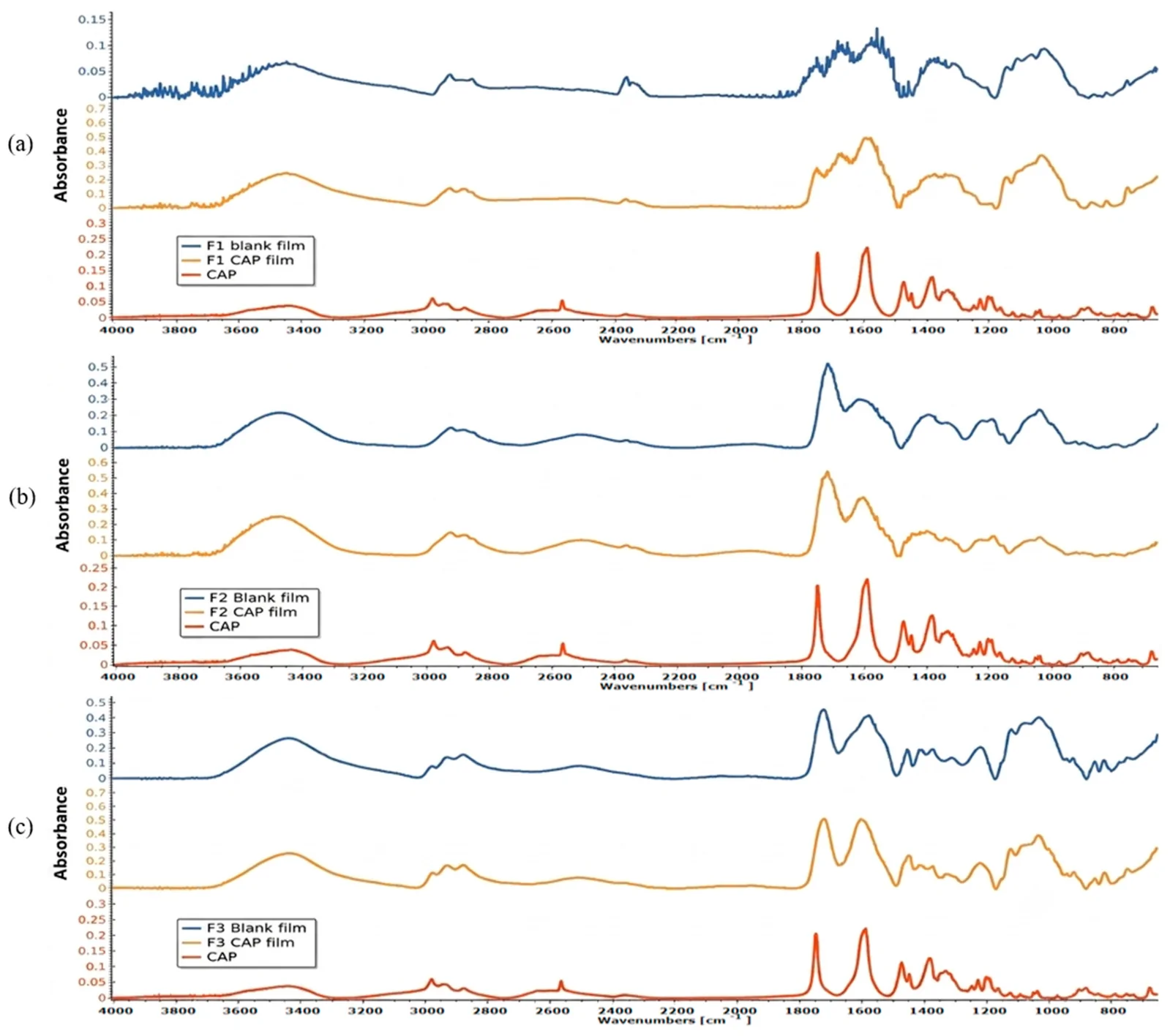
FT-IR spectra of pure CAP, F1 (**a**), F2 (**b**), and F3 (**c**) films.

**Figure 4:**
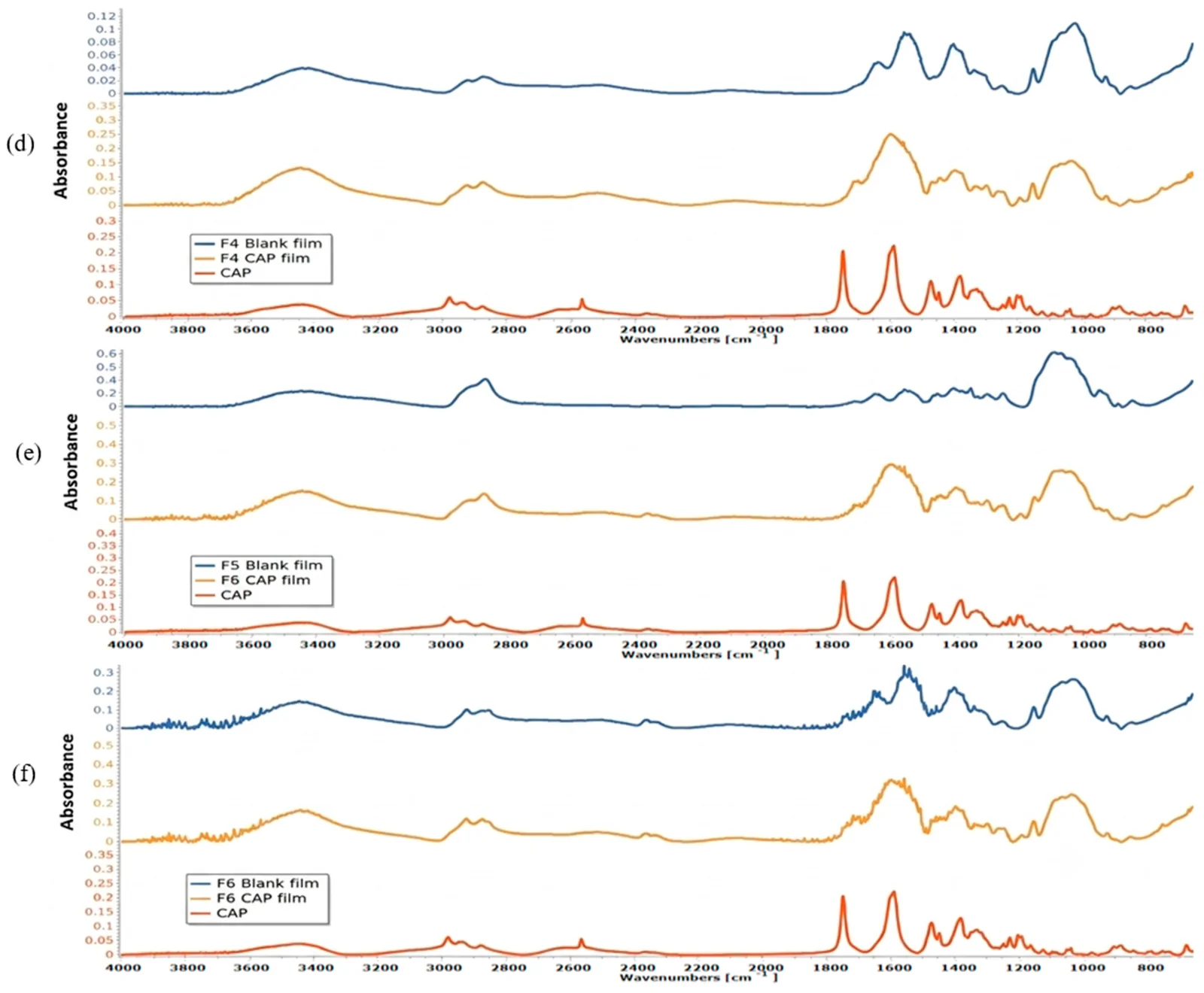
FT-IR spectra of pure CAP, F4 (**d**), F5 (**e**), and F6 (**f**) films.

**Figure 5:**
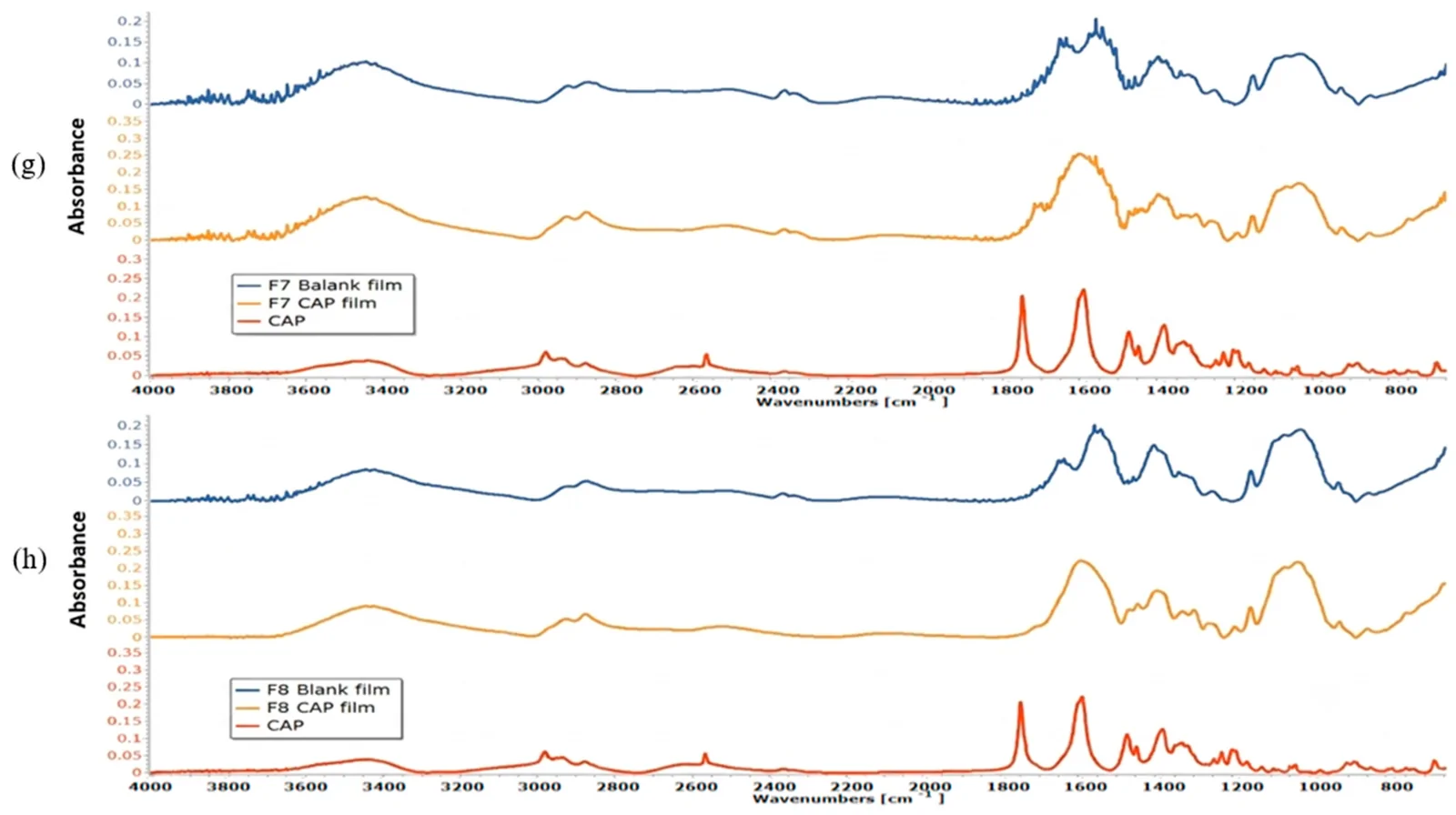
FT-IR spectra of pure CAP, F7 (**g**), and F8 (**h**) films.

The FT-IR spectra of raw CAP, blank films, and CAP-loaded films prepared using different acid variants (ascorbic acid, citric acid, lactic acid, and acetic acid) and with various additives (EDTA, PEG 400, SLS, and Span 20) are presented in Figure 3, Figure 4 and Figure 5.

In the raw CAP spectrum, the two bands at 2984 and 2880 cm^−1^ corresponded to the –CH_2_ and –CH_3_ stretching groups. A narrow band was observed at 2567 cm^−1^, which is characteristic of the –SH stretching group. The C=O vibration of the carboxyl group gave an intense band at 1750 cm^−1^, whereas an intense band at 1588 cm^−1^ indicated the C=O vibration of the amide group [43,44]. The diagnostic peaks of CAP were identifiable, although attenuated due to dilution within the polymer matrix, in the spectra of all drug-loaded films. This confirms successful and homogeneous incorporation of the active pharmaceutical ingredient (API) across all developed formulations.

All blank and drug-loaded films displayed broad O–H/N–H stretching vibrations of chitosan, acids, and glycerol in the 3400–3200 cm^−1^ region [24,45] and characteristic chitosan bands corresponding to amide I at ~1650 cm^−1^, amide II at ~1550 cm^−1^, and C–O–C glycosidic linkages at 1150–1000 cm^−1^. However, some peaks appeared broadened and slightly shifted in the F2 spectra [45].

In the drug-loaded films, peak broadening, slight shifts, and changes in intensity were observed, particularly in the carbonyl (1800–1600 cm^−1^), amine, and fingerprint regions. These spectral modifications indicate the formation of hydrogen bonds between the amino groups of chitosan, the carboxyl and thiol functionalities of captopril, glycerol, and the respective acids (ascorbic, citric, lactic, or acetic), as shown in Figure 3a–c and Figure 5h.

Characteristic bands related to the incorporated additives were also detected, further supporting their successful integration into the matrix: S=O asymmetric and symmetric stretching vibrations from SLS (~1220–1215 cm^−1^ and ~1085–1060 cm^−1^) (Figure 4d) [46]; strong C–O–C stretching from PEG 400 (~1100 cm^−1^) [47] and –CH asymmetric stretching (~2925 cm^−1^) and C=O stretching (~1740 cm^−1^) from Span 20 (Figure 4e,f) [48]; carboxylate asymmetric and symmetric stretching from EDTA (~1600–1580 cm^−1^ and ~1400 cm^−1^) (Figure 5g) [49]. Importantly, no new covalent peaks appeared, and no major functional group bands disappeared in any of the formulations. This demonstrates the physicochemical compatibility and chemical stability of CAP during the solvent-casting process, irrespective of the acid used or the presence of additives. Overall, the FT-IR spectroscopic analysis verifies that all investigated formulations provide a compatible and stable polymeric matrix suitable for the buccal delivery of captopril. The data support the successful integration of both the drug and the various excipients, with no evidence of chemical incompatibility.

### 3.8. Cytotoxicity Test

**Figure 6:**
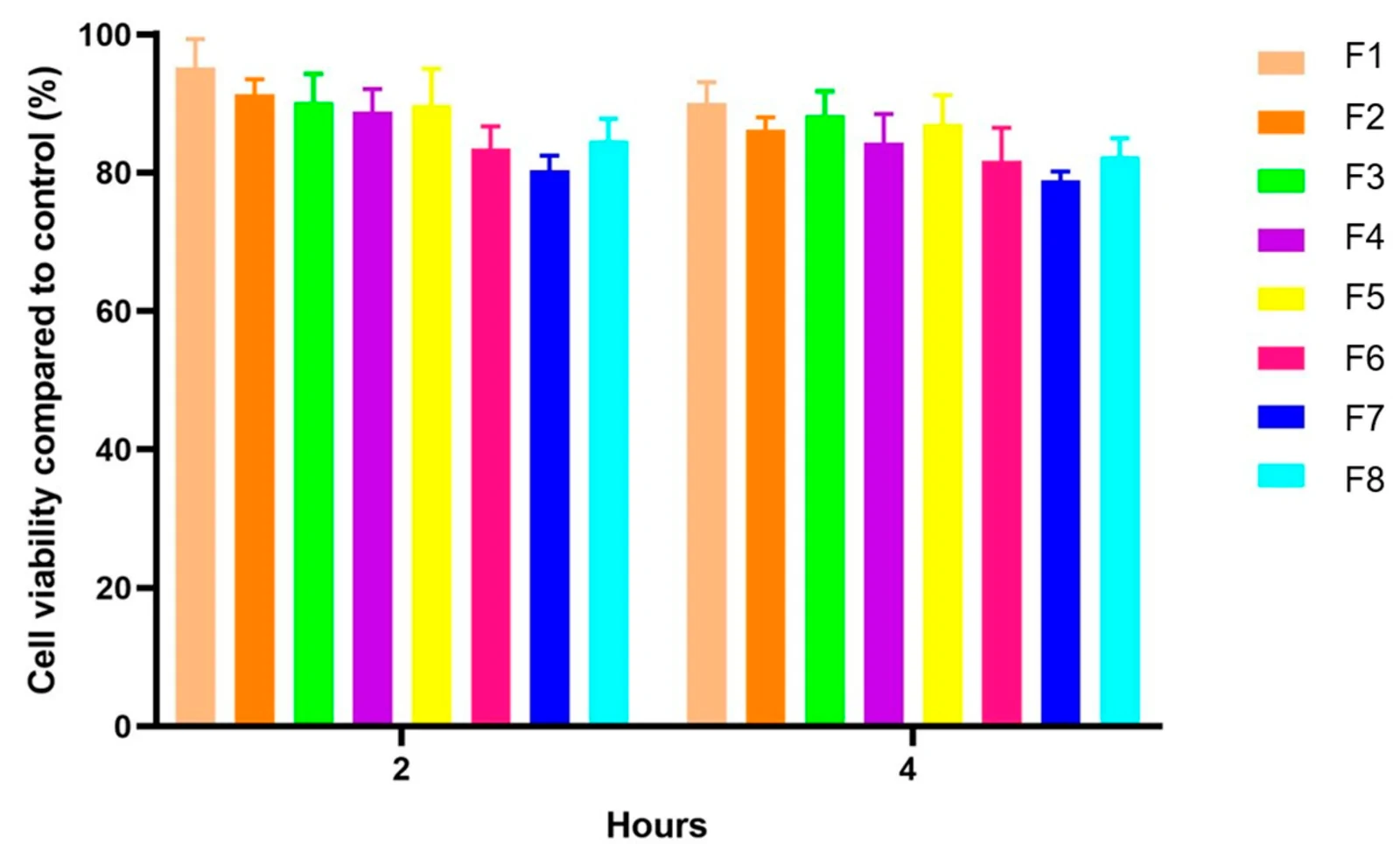
Cell viability of TR146 cells following 2 and 4 h of exposure to the films.

Evaluation of cytotoxicity is essential when investigating permeation enhancers because disruption of epithelial barrier integrity may be accompanied by cellular damage. Since TR146 cells are a well-established model of human buccal epithelium, the NR uptake assay provides relevant information regarding the compatibility of the developed formulations with buccal tissues. Neutral Red accumulates in the lysosomes of viable cells; therefore, reduced dye uptake reflects impairment of cellular membrane integrity, lysosomal function, or overall cell viability [50]. The cytotoxicity of the prepared chitosan-based buccal films containing captopril and various permeation enhancers was evaluated on TR146 cells using the Neutral Red (NR) uptake assay. Films were dissolved in Hank’s Balanced Salt Solution (HBSS) and applied to the cells for up to 4 h. Cell viability was expressed as a percentage of untreated controls (Figure 6).

No marked reduction in cell viability was observed between 2 and 4 h of exposure for any formulation, indicating that prolonged contact with the dissolved films did not substantially increase cellular damage. Furthermore, all formulations maintained cell viability greater than 81% compared to untreated control cells (100%) throughout the exposure period, indicating acceptable cytocompatibility under the experimental conditions [51]. No statistically significant differences were observed between the tested formulations or compared to the control at either time point (*p* > 0.05).

These findings align with previous reports on chitosan-based delivery systems. Chitosan itself and organic acid salts are widely recognized for their favorable biocompatibility profile in drug delivery applications [52,53]. Mild organic acids (e.g., ascorbic, citric, lactic, and acetic) are frequently used as solvents to dissolve or modify chitosan for buccal films, gels, or patches. These acids generally yield formulations with good biocompatibility. For instance, a recent study on chitosan hydrogels prepared with acetic, lactic, glutamic, and citric acids reported that all formulations maintained cell viability above 80% in cytotoxicity tests, confirming their suitability for applications [53]. Permeation enhancers, such as SLS, PEG 400, Span 20, and EDTA, are incorporated at low levels in chitosan-based buccal systems to improve drug penetration while minimizing irritation or cytotoxicity. The literature indicates that at appropriately low concentrations, these additives preserve cell viability in models like TR146 buccal epithelial cells [54].

Overall, all formulations maintained cell viability well above the 70% threshold commonly used to classify materials as non-cytotoxic in in vitro viability assays [55], demonstrating acceptable biocompatibility toward the TR146 cell line [51]. These results suggested that the selected permeation enhancers can be incorporated into chitosan buccal films without causing severe acute cytotoxicity during the intended application period, providing a strong indicator of suitability for further permeation studies.

### 3.9. In Vitro Permeation Test

**Figure 7:**
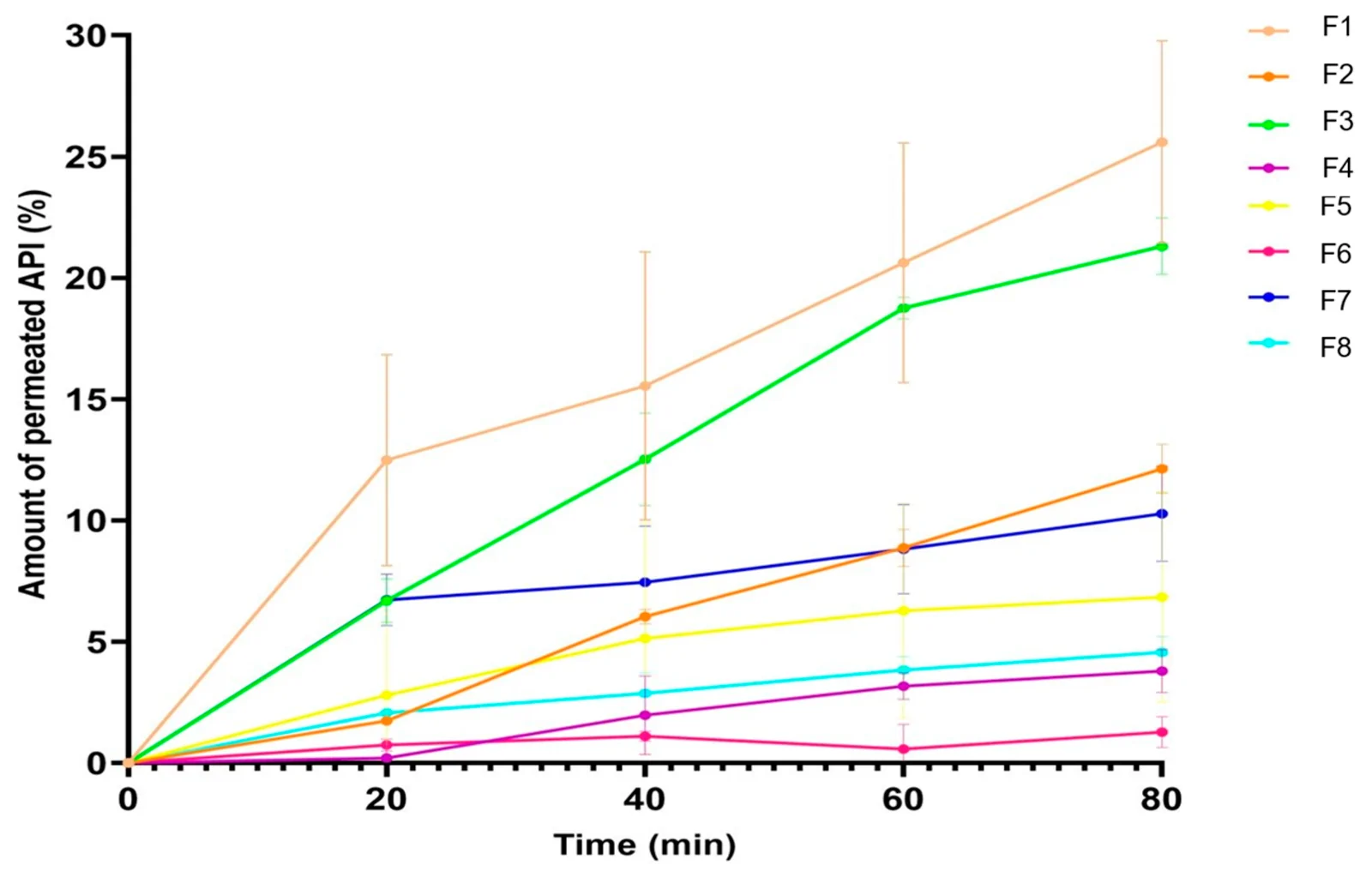
Amount of permeated CAP (%) from chitosan-based buccal films across TR146 cells (mean ± SD, *n* = 3). Reported data are as follows for 20, 40, 60 and 80 min. F1: 12.50% ± 4.6%; 15.55% ± 5.5%; 20.63% ± 4.9%; 25.60% ± 4.2%. F2: 1.74% ± 0.1%; 6.04% ± 0.3%; 8.87% ± 0.8%; 12.13% ± 1.0%. F3: 6.69% ± 0.9%; 12.53% ± 1.9%; 18.75% ± 0.4%; 21.30% ± 1.2%. F4: 0.21% ± 0.5%; 1.97% ± 1.6%; 3.17% ± 0.5%; 3.79% ± 0.9%. F5: 2.80% ± 3.9%; 5.14% ± 4.7%; 6.28% ± 4.4%; 6.85% ± 4.3%. F6: 0.74% ± 0.2%; 1.11% ± 0.2%; 0.57% 1.0%; 1.27% ± 0.6%. F7: 6.73% ± 1.1%; 7.45% ± 2.3%; 8.82 ± 1.8%; 10.28% ± 2.0%. F8: 2.07% ± 0.0%; 2.87% ± 0.8%; 3.83% ± 0.6%; 4.57% ± 0.6%.

The present study aimed to compare different chitosan salt forms and conventional permeation enhancers with respect to their ability to promote in vitro buccal drug transport. Accordingly, chitosan films prepared with various organic acids or established permeation-enhancing excipients were evaluated for their ability to enhance captopril transport across TR146 cell monolayers. Graphical results of the permeated amount of CAP are shown in Figure 7, while the corresponding average flux and apparent permeability values are shown in Table 4 and Table 5.

Compared with the in vitro drug dissolution test, where most formulations had similar dissolution profiles, in vitro permeability studies highlight the elevated amount of permeated CAP in the case of F1 and F3 formulations. These formulations also differ from the others, as their flux values are the highest at each time point. As F3 contained only lactic acid as the extra excipient, we consider this a possible alternative to ascorbic acid in buccal delivery. Formulations from F4 to F8 also contained acetic acid, and we observed a negative impact on their permeation.

This phenomenon can suggest that the difference between the in vitro dissolution and the cellular experiments is based upon the interaction between chitosan and the other excipients in each formulation. Chitosan itself is known to increase the permeability of metformin and insulin, among other APIs, in the case of buccal drug delivery systems [56,57]. Its permeability enhancer properties are mostly explained by the positive charges of the polymer, reorganizing tight junctions to improve permeation through the cell layers [52,58,59]. Permeation behavior is also influenced by the fact that chitosan can form hydrogels, which could remain intact during our experiments. The formation of stable hydrogels can explain the slow and limited permeation of captopril from certain formulations, despite the presence of surface-active agents like SLS or Span 20 [60,61]. In order to discriminate between the altered gelling behavior, which mostly impacts the release of the API from the hydrogel into the upper compartment of the cell culture insert, and the unfavorable interaction between cell surfaces and the chitosan, further studies are needed to examine the micro-rheology and the interaction of the molecules of the cell surface. However, it must be highlighted that the presence of seemingly inert excipients, such as acetic acid, can have a tremendous impact on the effects of formulations containing otherwise well-established permeation enhancers.

## 4. Conclusions

This work successfully formulated and characterized a series of chitosan-based mucoadhesive buccal films loaded with captopril as a model hydrophilic drug. The incorporation of different permeation enhancers, including organic acid salts of chitosan and conventional chemical enhancers, yielded films with uniform drug content, appropriate thickness, adequate mechanical strength, high folding endurance, and satisfactory mucoadhesive properties. All formulations exhibited rapid in vitro drug release, with more than 50% of captopril released within the first 15 min, which could be advantageous for the management of conditions requiring fast onset of action. Importantly, the films demonstrated good biocompatibility, maintaining TR146 cell viability above 81% after 4 h of exposure in the Neutral Red uptake assay. Among the investigated formulations, chitosan ascorbate-based films (F1) and chitosan lactate-based films (F3) showed the highest captopril transport across the TR146 cell model under the experimental conditions applied. These findings indicate that formulation composition, including the type of organic acid used for chitosan preparation, may influence captopril transport. However, the mechanisms underlying these differences could not be established in the present study and warrant further investigation. Overall, the findings demonstrate the potential of chitosan-based mucoadhesive films as a platform for buccal captopril delivery and highlight the importance of formulation composition in the development of such delivery systems.

## Figures and Tables

**Table 1:** Composition of the polymer film casting solution (*w*/*w*%). Distilled water was used as the solvent and added to a final weight of 100 g.

Sample No.	CHI	GLY	Asc. Acid	Cit. Acid	Lac. Acid	Ac. Acid	SLS	PEG400	Span 20	EDTA	CAP (mg/4 cm^2^)
F1	2	0.7	2.5	-	-	-	-	-	-	-	20
F2	2	0.7	-	2.5	-	-	-	-	-	-	20
F3	2	0.7	-	-	2.5	-	-	-	-	-	20
F4	2	0.7	-	-	-	2	0.011	-	-	-	20
F5	2	0.7	-	-	-	2		0.5	-	-	20
F6	2	0.7	-	-	-	2	-		0.2	-	20
F7	2	0.7	-	-	-	2	-	-		0.25	20
F8	2	0.7	-	-	-	2.5	-	-	-	-	20

**Table 2:** Physicochemical properties of the prepared films. Data are expressed as mean ± SD; *n* = 3; * *p* < 0.05.

Sample No.	Thickness (µm)	Moisture Content (%)	Breaking Hardness (N)	Mucoadhesion Force (N)	Puncture Resistance Time (s)	Folding Endurance	Drug Content (mg)
F1	231 ± 61 *	3.63 ± 0.36 *	26.64 ± 2.9 *	16.21 ± 1.63	10.22 ± 0.84 *	>300	21.5 ± 0.7
F2	270 ± 53 *	3.8 ± 0.3	15.14 ± 3.36 *	16.67 ± 2.13 *	10.39 ± 0.96 *	>300	20.1 ± 0.7 *
F3	228 ± 50 *	5.24 ± 1.47	25.2 ± 1.31 *	11.23 ± 1.71	13.78 ± 0.55 *	>300	21.6 ± 0.8
F4	164 ± 35	8.41 ± 1.12	42.17 ± 6.97	10.6 ± 1.72	12.74 ± 0.75 *	>300	21.5 ± 0.3 *
F5	181 ± 38 *	8.06 ± 0.97	35.28 ± 6.93	18.42 ± 2.42 *	14.16 ± 0.59	>300	17.4 ± 1
F6	187 ± 59	6.56 ± 1.66	48.15 ± 0.63 *	12.54 ± 1.33 *	11.59 ± 0.89 *	>300	20 ± 2.3 *
F7	183 ± 35	7.23 ± 1.46	48.17 ± 1.14	13.76 ± 1.86	12.25 ± 0.59	>300	22.2 ± 0.9
F8	173 ± 24	7.51 ± 0.91	45.7 ± 3.80	11.47 ± 2.18	9.17 ± 0.39	>300	19.4 ± 1.3

**Table 3:** Drug release kinetic parameters of CAP-loaded chitosan buccal films.

Sample No.	Zero Order R^2^	First Order R^2^	Higuchi R^2^	Korsmeyer–Peppas R^2^	n
F1	0.7505	0.9742	0.9307	0.9656	0.6623
F2	0.6567	0.9662	0.873	0.9984	0.4599
F3	0.7315	0.9711	0.9225	0.9945	0.547
F4	0.7249	0.9744	0.919	0.9999	0.4785
F5	0.6957	0.9489	0.9005	0.9991	0.5156
F6	0.5901	0.9885	0.7663	1	0.5691
F7	0.65	0.8995	0.8411	0.9999	0.4866
F8	0.7125	0.9947	0.9118	0.9931	0.4448

**Table 4:** Average flux (*J* in mg/min × cm^2^) of CAP from CHI-based buccal films across the TR146 cells.

Sample No.	20 min	40 min	60 min	80 min
F1	6.683×10^−3^	8.316 × 10^−3^	1.103 × 10^−2^	1.369 × 10^−2^
F2	9.315 × 10^−4^	3.228 × 10^−3^	4.744 × 10^−3^	6.486 × 10^−3^
F3	3.579 × 10^−3^	6.701 × 10^−3^	1.002 × 10^−2^	1.139 × 10^−2^
F4	1.115 × 10^−4^	1.056 × 10^−3^	1.694 × 10^−3^	2.029 × 10^−3^
F5	1.496 × 10^−3^	2.748 × 10^−3^	3.361 × 10^−3^	3.661 × 10^−3^
F6	3.971 × 10^−4^	5.940 × 10^−4^	3.060 × 10^−4^	6.812 × 10^−4^
F7	3.599 × 10^−3^	3.985 × 10^−3^	4.717 × 10^−3^	5.497 × 10^−3^
F8	1.108 × 10^−3^	1.536 × 10^−3^	2.048 × 10^−3^	2.442 × 10^−3^

**Table 5:** Apparent permeability (*P_app_* in cm/s) of CAP from CHI-based buccal films across the TR146 cells.

Sample No.	20 min	40 min	60 min	80 min
F1	3.155 × 10^−4^	3.926 × 10^−4^	5.208 × 10^−4^	6.465 × 10^−4^
F2	4.398 × 10^−5^	1.524 × 10^−4^	2.240 × 10^−4^	3.062 × 10^−4^
F3	1.689 × 10^−4^	3.163 × 10^−4^	4.735 × 10^−4^	5.379 × 10^−4^
F4	5.267 × 10^−6^	4.987 × 10^−5^	8.000 × 10^−5^	9.580 × 10^−5^
F5	7.066 × 10^−5^	1.297 × 10^−4^	1.587 × 10^−4^	1.728 × 10^−4^
F6	1.875 × 10^−5^	2.804 × 10^−5^	1.444 × 10^−5^	3.216 × 10^−5^
F7	1.699 × 10^−4^	1.881 × 10^−4^	2.227 × 10^−4^	2.595 × 10^−4^
F8	5.234 × 10^−5^	7.254 × 10^−5^	9.673 × 10^−5^	1.153 × 10^−4^

## Data Availability

Data are contained within the article.

## Abbreviations

The following abbreviations are used in this manuscript:

AC	Apical compartment
ACEI	Angiotensin-converting enzyme inhibitor
API	Active pharmaceutical ingredient
BC	Basolateral compartment
BCS	Biopharmaceutics Classification System
CAP	Captopril
CHI	Chitosan
CHI-Ac	Chitosan acetate
CHI-Asc	Chitosan ascorbate
CHI-Cit	Chitosan citrate
CHI-Lac	Chitosan lactate
FT-IR	Fourier transform infrared spectroscopy
GLY	Glycerol
HATR	Horizontal attenuated total reflectance
HBSS	Hank’s balanced salt solution
LOD	Limit of detection
LOQ	Limit of quantification
NR	Neutral Red
PB	Phosphate buffer
Ph.Eur.	European Pharmacopeia
TEER	Transepithelial electrical resistance
SD	Standard deviation
